# Performing Platform Governance: Facebook and the Stage Management of Data Relations

**DOI:** 10.1007/s11948-024-00473-5

**Published:** 2024-04-04

**Authors:** Karen Huang, P. M. Krafft

**Affiliations:** 1 McCourt School of Public Policy, Georgetown University, Washington, DC USA; 2 Creative Computing Institute, University of the Arts London, London, UK

**Keywords:** Data relations, Platform governance, Controversy, Reflexivity, Performativity

## Abstract

Controversies surrounding social media platforms have provided opportunities for institutional reflexivity amongst users and regulators on how to understand and govern platforms. Amidst contestation, platform companies have continued to enact projects that draw upon existing modes of privatized governance. We investigate how social media companies have attempted to achieve closure by continuing to set the terms around platform governance. We investigate two projects implemented by Facebook (Meta)—authenticity regulation and privacy controls—in response to the Russian Interference and Cambridge Analytica controversies surrounding the 2016 U.S. Presidential Election. Drawing on Goffman’s metaphor of stage management, we analyze the techniques deployed by Facebook to reinforce a division between what is visible and invisible to the user experience. These platform governance projects propose to act upon *front-stage data relations:* information that users can see from other users—whether that is content that users can see from “bad actors”, or information that other users can see about oneself. At the same time, these projects relegate *back-stage data relations*—information flows between users constituted by recommendation and targeted advertising systems—to invisibility and inaction. As such, Facebook renders the user experience actionable for governance, while foreclosing governance of back-stage data relations central to the economic value of the platform. As social media companies continue to perform platform governance projects following controversies, our paper invites reflection on the politics of these projects. By destabilizing the boundaries drawn by platform companies, we open space for continuous reflexivity on how platforms should be understood and governed.

## Introduction

For Facebook^1^ users, several noticeable features may currently be visible on the user interface. A “Privacy Checkup” tool—represented by a lock and floating heart—guides the user through “Who can see what you share” and “Your ad preferences on Facebook.” A series of pages will show the user how to disable which advertisers can reach them with a click of a button, leading to a large reassuring checkmark of “You’re All Set”. In the Accounts Center, a user can also delve more extensively into their “Ad preferences”, selecting “Categories used to reach you” by advertisers, such as birth month or household income.

A page detailing the platform’s Community Standards reads, “Meta recognizes how important it is for Facebook to be a place where people feel empowered to communicate, and we take our role seriously in keeping abuse off the service. That’s why we developed standards for what is and isn’t allowed on Facebook.” The company emphasizes, “Our commitment to expression is paramount, but we recognize the internet creates new and increased opportunities for abuse.” Referencing one of the values included in the Community Standards, the company assures: “We want to make sure the content people see on Facebook is authentic” (Transparency Center, n.d.).

From this user view, a display of governance initiatives appears on the Facebook platform: Facebook seems to have made some substantial efforts to provide user controls over how advertisers can reach them, and to ensure that users see only content that meets Community Standards. These efforts reflect the company’s expansion of governance projects in response to their involvement in a series of high-profile controversies, from the emotion manipulation experiment in 2014 to Cambridge Analytica and Russian interference in 2016 to the January 6th, 2021 insurrection.

Although this user view might be reassuring, ongoing controversies reveal much more going on—and remaining unchanged—behind the scenes. In October 2021, whistleblower Frances Haugen leaked internal Facebook documents showing the extent to which the company’s operations and technical infrastructure depart from the platform’s front-stage presentation. As Haugen stated in her testimony to the U.S. Senate Committee on Commerce, Science and Transportation, “I came forward because I recognized a frightening truth: almost no one outside of Facebook knows what happens inside Facebook” (Haugen, 2021d).

In this paper, we analyze how social media platforms like Facebook perform platform governance following controversy. By investigating the techniques deployed to reinforce a division between what is visible and invisible to the user experience, we illustrate how these platform governance projects focus on making modifications to the user view while obscuring the information flows central to the economic value of the platform. As such, we show how these projects normalize the governance of the user experience while foreclosing governance of the political economy of platforms. As social media companies continue to perform platform governance projects following controversies, our paper invites reflection on the politics of these projects. By destabilizing the boundaries drawn by platform companies, this analysis aims to open space for continuous reflexivity on how to understand and govern platforms.

## Institutional Reflexivity on Platform Governance

Platform companies have consistently played a prominent role in shaping how their technologies are understood for governance by setting the terms of the discourse (Gillespie, 2010, 2018, 2023; Hoffmann et al., 2018) and via technical design choices (DeNardis, 2012; DeNardis & Hackl, 2015). Governance *by* platforms refers to governance mechanisms implemented directly in platforms, usually by the companies that own them, situated within broader regulatory regimes (Gorwa, 2019). The privatization of speech regulation via content moderation (Gillespie, 2010, 2018; Klonick, 2018), and the privatization of conditions of privacy via the technical affordances of platforms, are two key ways in which platform companies enact governance (DeNardis & Hackl, 2015).

Platform companies’ strategic term “platform” has stabilized how these technologies are understood and regulated as neutral information intermediaries protected from liability for content generated by users (Gillespie, 2010). As Gillespie (2010) explains: “A term like ‘platform’ does not drop from the sky […] These are efforts […] to make claims about what these technologies are and are not […] to establish the very criteria by which these technologies will be judged, built directly into the terms by which we know them” (p. 359). Section 230 of the Communications Decency Act dubbed internet platforms as “providers of ‘interactive computer services’”, not as publishers or speakers of content posted by users on the platform’s website (Klonick, 2018, p. 1604). Under the safe harbor protections of statutes like Sect. 230, platform companies have drawn upon legal framings of these technologies as “neutral tools that are, and should be, exempt from more intrusive oversight” (Cohen, 2020, p. 654), and have normalized content moderation—the practice of monitoring, reviewing, and taking action against content deemed as harmful, abusive, or misleading—as the dominant mode of platform governance (Gillespie, 2018; Klonick, 2018). From the user view of the platform, content moderation provides guidelines on which posts, advertisements, news stories, and other content appear on the platform.

Social media companies also play a direct role in governance through their technical design affordances of online privacy policies. Extant digital-privacy law^2^ in the United States emphasizes the importance of an individual user’s control over their data. Under notice-and-consent privacy practices enforced by the Federal Trade Commission (FTC), platforms are required to notify users of data collection and handling practices via contractual terms of service, and users can consent to those terms of service. Under the extant notice-and-consent privacy regime, also called transparency-and-choice (Nissenbaum, 2011), a platform’s terms of service and interface design choices serve as a form of privatized platform governance (DeNardis & Hackl, 2015). As Nissenbaum (2011) explains, “Transparency-and-choice appears to model control because it allows individuals to evaluate options deliberately and then decide freely whether to give or withhold consent” (p. 34). However, the technological affordances of platforms are often designed in ways that could constrain opting out, and behavioral insights deployed on platforms could steer users to consent to the terms of service (Brignull, 2013; Susser et al., 2019; Viljoen, 2021). Advocates who nevertheless want to maintain this paradigm of transparency-and-choice propose practices to increase transparency, such as “stipulating shorter policies that are easier to follow, along the lines of nutritional labels”, or for framing choices as “opt in” rather than “opt out” (Nissenbaum, 2011, p. 35). From the user view of the platform, these transparency efforts would mean shorter and easier-to-read privacy policies, before the user clicks “Agree” and proceeds to use the platform.

These extant modes of privatized governance support how platform companies aim for platforms to be understood and governed: as neutral information intermediaries, and as social networking sites that offer a way of connecting family and friends in exchange for users’ acceptance of the terms of service.

### Controversy and Reflexivity

In recent years, social media companies have faced continuous and high-profile controversies around the role that platforms play in U.S. political processes such as elections. These controversies have spanned a range of concerns from platforms’ exploitation of user data for political campaigns, to regulation of speech, to online spreading of misinformation, thus sparking reflection and scrutiny amongst users, regulators, and broader publics.

Controversies surrounding the 2016 U.S. Presidential Election—specifically, Russian interference and Cambridge Analytica—marked a turning point in terms of public outrage. Media coverage in March 2018 brought the controversies to widespread attention (e.g., Cadwalladr & Graham-Harrison, 2018; Rosenberg et al., 2018). The hashtag #deleteFacebook went viral on Twitter, as Facebook users expressed frustration over lack of user data protection (Khosrowshahi & Mitra, 2018). Mark Zuckerberg’s testimony in the Senate in April 2018—also widely covered in the news media (e.g. “Transcript of Mark Zuckerberg’s Senate Hearing,” 2018; Watson, 2018)—marked increased scrutiny regarding the governance of platforms.

In the wake of the 2020 U.S. Presidential Election, Facebook and Twitter’s suspension of former President Donald Trump’s account led to the further realization of the extent to which these platforms have control over public discourse. Following the U.S. Capitol insurrection on January 6th, 2021, Facebook announced a ban of Trump’s account, citing “use of our platform to incite violent insurrection against a democratically elected government” (Rosen, 2021). Twitter’s ban of Trump, and a slew of platform companies blocking content by Trump and Trump’s supporters, led to responses from publics and policymakers concerning platform companies’ role in the governance of speech (e.g. Anderson, 2019; Douek, 2019). In March 2021, Zuckerberg testified before the U.S. Congress yet again (Zuckerberg, 2021a), where he faced hard-hitting questions about the role of Facebook in elections.

People are normalized to understanding platforms as ways to share and see content from friends and followers. However, these controversies have continuously revealed that platforms are also something else—that there is a whole host of operations going on behind the scenes. Public outrage reflects a shift in recognition that these platforms prove to be more than meets the eye. After a controversy in 2014 involving Facebook’s emotion contagion experiment—which revealed how Facebook had tweaked the presentation of content on its News Feed in order to measure how interface design could manipulate user emotions (Kramer et al., 2014)—Tufekci (2014) explained that publics’ “broad and negative reaction suggests that algorithmic manipulation generates discomfort exactly because it is opaque […] in an environment of information asymmetry.” Such controversies revealed how platform companies like Facebook are continuously shaping a digital sociality (Marres, 2017)—that is, they are “engineering the public”—out of view (Tufekci, 2014).

These controversies have pushed regulators to reevaluate their understanding of platforms and to rethink platform governance. How regulators conceive of platforms was famously revealed during Senator Orrin Hatch’s question for Mark Zuckerberg during the April 2018 testimony: asking Zuckerberg, “so, how do you sustain a business model in which users do not pay for your service?”, Zuckerberg responded, “Senator, we run ads” (*S.Hrg. 115–683—Facebook, Social Media Privacy, and The Use and Abuse of Data*, 2018). As Frances Haugen explained in October 2021 in her written testimony to the U.S. Senate Committee on Commerce, Science and Transportation, regulators only have a limited view of platforms like Facebook, since Facebook “hides behind walls that keep the eyes of researchers and regulators from understanding the true dynamics of the system” (Haugen, 2021d). She urged, “The severity of this crisis demands that we break out of previous regulatory frames. Tweaks to outdated privacy protections or changes to Sect. 230 will not be sufficient” (Haugen, 2021d).

At the heart of these controversies was the realization that how platforms aim to be understood—for example, as neutral information intermediaries—was becoming destabilized. Controversies surrounding platforms have provided opportunities for institutional reflexivity amongst publics, users, and regulators on the terms by which to understand platforms, and how to govern them. Institutional reflexivity involves systematic reflection on ethical, economic, social, and political commitments underlying science and technology and their governance (Stilgoe et al., 2013; Wynne, 2011). In contrast to reflexivity defined as private reflection, critique, and interventions among scientists in their own communities, institutional reflexivity includes wider societal engagement of groups such as publics, regulators, users, research funders, and other institutions of democratic governance (Schuurbiers, 2011; Stilgoe et al., 2013; Wynne, 1993, 2011). In the context of platform governance examined in this paper, institutional reflexivity refers to reflection and critique of social media platforms primarily by users and regulators.

## Stabilizing Understandings of Platforms and Platform Governance

Platform governance involves a complex network of interactions amongst various actors (e.g., corporate, state, and non-state) organizing, structuring, and regulating private information intermediaries (Gorwa, 2019). Platform governance—which involves both governance *by* platforms, and governance *of* platforms (Gorwa, 2019)—takes on many different modes. How to understand and govern platforms remains contested, underdetermined, and ongoing (Gillespie, 2023; Gillespie et al., 2020; Gorwa, 2019; Klonick, 2018; Napoli & Caplan, 2017). For example, Klonick (2018) explains how competing analogies for these technologies—such as editorial desks, forums, public utilities, or “new governors”—influence how they ought to be regulated.

As users and regulators have engaged in projects of reflexivity in response to controversies, platform companies have doubled-down on their own governance projects. Examples of these initiatives include Facebook’s creation of an Oversight Board (Clegg, 2020), authenticity regulation involving the removal of fake accounts (Haan, 2020), and increased transparency regarding the control of information (Ananny & Crawford, 2018; Gorwa & Ash, 2020). These privatized governance projects, through their diagnoses and proposed actions, reify how platform companies want users and regulators to understand their platforms. As Gillespie (2023) notes, in the past two decades, platform companies have responded to controversies by developing efforts that “help to stabilize the understanding of the problems at hand, valorize the role of those companies in addressing them, demarcate appropriate solutions, and normalize the relations between the company, public, market, and state on which they depend” (p. 406).

Following Science and Technology Studies (STS) research on closure, we define closure as the stabilization of a debate or a technological artifact (e.g. Hilgartner, 2000; Pinch & Bijker, 1984). In the midst of users and regulators engaging in projects of reflexivity, platform companies like Facebook have also attempted to achieve closure after controversy. Critical data studies and STS scholars have pointed to industry capture of critique and reflection on digital technologies (e.g. Frahm et al., 2022; Gillespie, 2023; Whittaker, 2021), leading to initiatives subsumed by corporate logics (e.g. Metcalf et al., 2019). As new forms of knowledge are generated by ethics and governance projects (Finn & Shilton, 2023), it is important to critically examine the work done by these projects.

We investigate how social media companies like Facebook may attempt to continue setting the terms around platform governance. By analyzing these performative attempts, we call attention to how they could obstruct processes of institutional reflexivity. This analysis aims to serve reflexivity amongst users, regulators, and broader publics to continuously re-imagine the institutional arrangements of platform governance. Maintaining such processes contributes to the overall project of democratic governance of science and technology, which requires continuous re-making. As Wynne (2011) argues, “In a democratic society which needs a healthy and responsive, versatile science, this enhanced sensitivity and attunement to democratic forces and needs is a crucial quality which cannot be taken for granted, nor can it ever be finally designed; but it must be worked at, continually” (p. 793).

As mentioned above, the privatization of speech regulation via content moderation (Klonick, 2018) and the privatization of conditions of privacy via the technological affordances of platforms—which pertain to the regulation of communicative expression—are two key ways in which platforms enact governance (DeNardis & Hackl, 2015). In this paper, we analyze two case studies of platform governance enacted by Facebook in response to controversies surrounding the 2016 U.S. Presidential Election. In response to the Russian Interference controversy, Facebook expanded its project on authenticity regulation^3^ as part of their broader efforts to govern platform speech (Haan, 2020). In response to the Cambridge Analytica controversy, Facebook developed and expanded projects on privacy controls, which draw from extant platform governance frameworks focused on transparency (Gorwa & Ash, 2020), and specifically the transparency-and-control approach to privacy (Nissenbaum, 2011). In our analyses, we show how Facebook has attempted to achieve closure—that is, to stabilize the terms of debate—through these platform governance projects. Our guiding research questions are the following: How do Facebook’s projects of authenticity regulation and privacy controls attempt to shape how platforms are understood for governance? How do these projects aim to draw the boundaries of platform governance?

## Maintaining the Front-Stage, Back-Stage Division

Platform companies deploy discursive strategies to normalize how platforms are understood (Gillespie, 2010), as they attempt to achieve a desired impression with users and regulators. To mobilize our analysis of how Facebook attempts to achieve closure on how its platform is understood for governance, we draw upon a dramaturgical perspective.

The sociologist Erving Goffman (1956) investigated how individuals selectively reveal and conceal information to create a desired impression with an audience. Participants in social interactions self-consciously—that is, strategically, with awareness of what they reveal and conceal—create and maintain impressions for their intended audiences. As Hilgartner (2000) explains, “Goffman’s point […] is that the participants in social interactions experience an active, theatrical self-consciousness. They are well aware that their actions create impressions and—like actors onstage—use a range of dramatic devices to create and maintain appearances” (p. 8). Hilgartner (2000) extended Goffman’s dramaturgical perspective to STS, analyzing how institutions such as the National Academy of Sciences selectively reveal and conceal information in order to achieve credibility with audiences. A dramaturgical perspective invites analysis of techniques utilized by platform companies like Facebook to normalize how platforms are understood for governance.

Stage management is a device for achieving closure. As Hilgartner (2000) explains, “Stage management is a technology of closure, and local systems and conditions that shape collective modes of information control are an important part of the social processes that shape the production of knowledge” (p. 149). In Goffman’s concept of “region behavior”, a front region (front stage) is perceived by an audience, and a back region (back stage) is hidden from that audience. In Goffman’s analysis, the separation between the front stage and back stage is essential for maintaining a successful performance—meaning that the audience believes in the performance.

Goffman’s concept of region behavior is well-suited to analyzing strategies deployed by Facebook to persuade users and regulators. In the context of our analysis, we designate the front stage as what is visible to the user experience, and the back stage as what is outside the user experience. The Facebook platform is structured by a division between a front region visible to users, and a back region invisible to users. Computer scientists and web designers rely explicitly on a division between what they call “front-end” versus “back-end” design (e.g. Koleva et al., 2015; Smith, 2012). Front-end design encapsulates the “user experience (UX)” and the software that directly interfaces with users. In contrast, “back-end” software is everything else in the “software stack” that is literally and conceptually hidden from users. “Front-end” software predominantly consists of the code that web browsers interpret in order to display websites on users’ digital screens. Front-end design thus focuses on aspects of “user interaction”, “accessibility”, “usability”, “privacy controls”, platform elements, functionality, etc. “Back-end” software includes database queries, optimization for efficient code execution, internal tooling, the machinery of targeted advertising, etc. The front-stage, back-stage metaphor characterizes the division between visible content and underlying technical infrastructure (DeNardis, 2012; DeNardis & Hackl, 2015). The front-stage, back-stage metaphor also corresponds to Facebook’s organizational structure, as a large tech company organizes its teams and divisions (e.g., public relations) in part by front- versus back-end considerations (Feitelson et al., 2013) As such, the front-stage, back-stage metaphor reflects the architecture of software systems that constitute Facebook’s platform, as well as Facebook’s organizational structure.

Boundary-drawing refers to practices of demarcation in the pursuit of authority and material resources (Gieryn, 1983). Most users and regulators form an understanding of the Facebook platform through the user interface—that is, through the user view. Analyzing the politics of data assemblages, Jasanoff (2017) has shown how what is made visible gets rendered into what is actionable for governance. We examine how platform companies take advantage of what is visible to the user experience to draw the boundaries of platform governance. In the context of our two case studies, stage management refers to the *maintenance* of a division between the front stage and back stage. In what we call the *stage management of data relations,* we analyze the techniques deployed by Facebook to reinforce a division between what is visible and what is invisible to the user experience, in their efforts to draw the boundaries of platform governance.

### Stage Management of Data Relations

Facebook’s platform governance projects enact diagnoses and proposals for action that appear within the user view of the platform—that is, they depict a set of relations between the user and some other actor intuitive to the user experience. We call these *front-stage data relations:* data relations^4^ that are visible to the user experience of the platform. Examples of Facebook’s front-stage data relations include “Facebook friends”, messages exchanged between users, and Facebook groups, as well as one user looking up another user’s page, or one user creating a post that is added to another’s timeline. These front-stage data relations capture how users’ interactions on social media sites such as Facebook continuously configure how “friendship” is experienced and defined in both online and offline contexts (Marres, 2017).

At the same time, Facebook’s governance projects occlude data relations that are outside of the user’s experience of the platform, which we describe as *back-stage data relations.* Our conception of back-stage data relations closely resembles what both Couldry and Mejias (2019) and Viljoen (2021) conceptualize as data relations that drive economic value. Facebook’s back-stage data relations include transmission of user data to specific advertisers, relations between users based on optimized metrics (e.g., in the context of recommendation system algorithms), relations between users induced by statistical inference involved in advertising algorithms (cf., horizontal data relations, Viljoen, 2021), and relations between users and the platform whereby users’ relevant demographic data are extracted and stored (cf., vertical data relations, Viljoen, 2021). An extensive literature on the political economy of data exposes the everyday extraction of data as capital (Sadowski, 2019) under “surveillance capitalism” (Zuboff, 2015). Since Facebook’s economic value derives from the commodification of social relations for the advertising market (Arvidsson, 2016), Facebook’s back-stage data relations drive the economic value of the Facebook platform.

In each of our case studies, we show how Facebook governs data relations on the front stage, and relegates data relations outside of the user experience to invisibility and inaction. We show how Facebook draws the boundaries of platform governance around modifying the user experience of the platform, while occluding data relations that drive the economic value of the platform. Through this critical inquiry, we question the normalization of platform governance as governance of the user experience.

## Authenticity Regulation

### Governing Front-Stage “Bad Actors”

This case details a governance project that Facebook has reinforced and expanded in response to Russian Interference in the 2016 U.S. Presidential Election. In brief, in the years leading up to the election, a collection of accounts operated by the Internet Research Agency (IRA), a Russian “troll farm”, generated and disseminated content across a variety of platforms, including Facebook, Twitter, and YouTube, in order to foment anger, suppress voter participation, increase political polarization, and support the election of Donald Trump (Abrams, 2019; Dilanian, 2019).

Facebook executives first started publicizing the term “bad actor” in the summer of 2017 (Haan, 2020). After the company learned of Russian interference, Facebook identified the IRA as the “bad actor” that “has consistently used inauthentic accounts to deceive and manipulate people” (Stamos, 2018). Facebook executives Sheryl Sandberg and Mark Zuckerberg increasingly deployed the terms “bad actors” and “adversaries” to refer to inauthentic accounts that “abuse” the platform and engage in “coordinated inauthentic behavior” (Haan, 2020).

Facebook drew upon prevailing discourses surrounding Sect. 230’s granting of platforms as neutral intermediaries. Roger McNamee, an early investor in Facebook and an advisor to Mark Zuckerberg, upon warning Zuckerberg and Sandberg of “bad actors […] taking the tools created for advertisers and using them to harm innocent people”, recounted how they “were just determined to hide behind the legal notion that they were a platform, not a media company and therefore not responsible for what third parties did” (PBS NewsHour, 2018).

Facebook maintained a distinction between appropriate and inappropriate use of its platform—Facebook the platform is neutral but can be used in “good” or “bad” ways. “Bad actors” are those who “abuse” the platform, while implied “good actors” are those who use the platform in proper ways. For instance, in a Facebook post directed toward users, Zuckerberg (2018b) explained, “When people can connect with each other, they can build communities around shared interests wherever they live in the world. But we've also seen how people can abuse our services, including during elections.” Similarly, in another post, Zuckerberg (2018c) lamented that since the 2016 election, “One of the most painful lessons I've learned is that when you connect two billion people, you will see all the beauty and ugliness of humanity.”

Since Facebook users experience the platform as the sharing of content with other users, Facebook paints a picture visible and intuitive to the user experience: individual users are presented with content served by the Facebook platform, and this content was generated by either “good” or “bad” actors. To illustrate an example, a user in a conservative Facebook group may see a post with a Pepe the Frog meme, posted by a user account associated with the IRA (the “bad actor”). Indeed, Zuckerberg explained at the April 2018 Senate Hearing: “approximately 126 million people may have been served content from a Facebook Page associated with the IRA” which “generated around 80,000 Facebook posts over about a 2-year period” (*S.Hrg. 115–683—Facebook, Social Media Privacy, and The Use and Abuse of Data*, 2018).

Facebook maintained that “bad actors” were the source of problematic content. Indeed, Facebook and other social media companies “commonly elide the difference between authentic *identity* and authentic *content*, suggesting that people who present only true information about themselves produce authentic (i.e., good) speech” (Haan, 2020, p. 628). Zuckerberg pointed to fake accounts as “the source of much of the spam, misinformation, and coordinated information campaigns” (Zuckerberg, 2018c), and stated, “Fake accounts are one of the primary vehicles for spreading misinformation—especially politically-motivated misinformation and propaganda” (Zuckerberg, 2018b). As such, Facebook sustained a discourse around “bad actors” as liable for problematic content on the platform.

Facebook’s authenticity regulation project puts the data relation between the user and “bad actor” within the sphere of governance. Having established that “bad actors” are liable for problematic content, Facebook expanded authenticity regulation to address those “bad actors”. First, Facebook proposed to remove inauthentic accounts. Zuckerberg (2018c) explained to users that “the most effective way to stop the spread of misinformation is to remove the fake accounts that generate it.” At the April 2018 Senate Hearing, Zuckerberg repeatedly assured: “Since 2016 […] we’ve built more advanced AI tools to remove fake accounts more generally” (*S.Hrg. 115–683—Facebook, Social Media Privacy, and The Use and Abuse of Data*, 2018). Furthermore, Zuckerberg (2018b) insisted to users that Facebook was taking down the IRA’s network of pages and accounts, and, “With advances in machine learning, we have now built systems that block millions of fake accounts every day.”

In addition, Facebook expanded its identity verification rules for users. In April 2018 Facebook initiated a mandatory identity verification process for all advertisers who want to run political or issue ads (Haan, 2020). Zuckerberg explained, “To get authorized, advertisers will need to confirm their identity and location” (*S.Hrg. 115–683—Facebook, Social Media Privacy, and The Use and Abuse of Data*, 2018). In May 2018, Facebook expanded its identity verification process to require “paid for by” labels on all political and issue ads for any publisher of paid content (Constine, 2018).

Furthermore, Facebook updated their “Community Standards”—their guidelines for content moderation—to encompass these actions against “bad actors”. “Authenticity” as a value was officially added to Facebook’s Community Standards in 2019 (Haan, 2020). As an expansion of its content moderation efforts, Facebook’s authenticity regulation project aims to take action on problematic content by targeting the creators of that content.

**Fig. 1:**
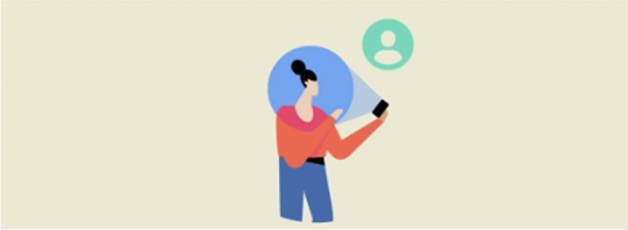
Screenshot of Depiction of “Authenticity” for the User (Transparency Center, n.d.)

Facebook’s authenticity regulation project—and corresponding actions against “bad actors”—constitute a front-stage performance for the user. Given that a piece of content is generated by a “bad actor”, removing that “bad actor” removes the content from the user view. To help users intuitively understand identity verification checks, Facebook even created a compelling illustration: “A graphic embedded in Facebook’s Community Standards webpage visually depicts ‘Authenticity’ as an expressionless woman shining a light on her own face while a figure watches” (Haan, 2020, p. 664) (see Fig. 1, Transparency Center, n.d.). A page titled, “Authenticity Matters: the IRA Has No Place on Facebook'' provides screenshots of Facebook page posts and Facebook ads generated by the IRA, overshadowed by a headline graphic highlighting how many of these accounts and pages have already been taken down by Facebook (Stamos, 2018).

By persuading users to intuitively understand the problem of “bad actors” and see for themselves the solution on the user interface (e.g., taking action against “bad actors”), Facebook attempts to normalize the governance of “authenticity”. Facebook puts on a front-stage performance of taking action on data relations visible to the user experience. In the midst of these efforts, Facebook correspondingly also relegates data relations outside of the user experience to invisibility and inaction (cf., Jasanoff, 2017).

### Occluding Back-Stage Data Relations

The front-stage picture hides from users and regulators a highly complex network of data relations. These back-stage data relations are constituted by Facebook’s recommendation and ranking systems central to Facebook’s business operations. The core function of these systems constitutes deciding which posts and pages to show to Facebook users, and in which order of presentation (Mac, 2021). In terms of economic relevance for Facebook, advertising space at the top of its pages can be sold for higher amounts because ads in those places will be more likely to be seen and clicked. In addition, effective ranking and recommendation is core to keeping users on the platform, such that there are more opportunities for those users to view and click on ads (Vaidhyanathan, 2018). How Facebook intentionally shapes its rankings to manipulate users has been central to several controversies (Kramer et al., 2014; Zuboff, 2015), but further details of the back-stage data relations situated within Facebook’s recommendation systems were revealed in the Facebook Papers leak by Frances Haugen. We illustrate an example of a back-stage data relation exposed in this leak: the relation between users and other users, structured by the metric of MSI (“meaningful social interaction”).

MSI is a metric that structures a relation between two entities. Facebook defines MSI as: “‘[A]ll interactions between two users where the initiator is not the same as receiver (e.g. a like on a friend reshare, or a comment reply to a user's comment on a public post)’” (Haugen, 2021b, p. 2). Two users who are already in relation with each other, specifically via a front-stage data relation of seeing each other’s posts or comments, are also in an additional data relation constructed by Facebook’s back-end operations. MSI is an aggregate of counts of interactions between the user and other users on Facebook—for instance, it captures how many times a user has replied to a friend’s posts, and how many times that user has interacted with other users’ posts. The relation between two users, where one user might see another user’s posts or comments, is numerically structured by the metric of MSI.

Facebook’s recommendation systems optimize for expected MSI. In order to rank different pieces of content (e.g., posts), Facebook’s recommendation systems will compute a predicted MSI score and put those posts with the highest predicted score at the top of a user’s newsfeed (Mac, 2021). An algorithm revealed in the Facebook Papers, “d_share_msi_score”, aims to predict content that users are likely to engage with, operationalized as the number of posts, likes, reshares, replies, and so on: “the way such content creators can contribute to MSI is by posting content that you might reshare for your friends to engage on or reshare themselves. This is precisely what we predict and uprank via d_share_msi_score"” (Haugen, 2021b, p. 7). Because the algorithm optimizes for expected MSI, the algorithm optimizes for that content to be engaged with or reshared by other users. The documents explain, “‘Downstream MSI’ is the process by which: a user posts content, then it gets shown to a viewer using an algorithm (d_share_msi_score), who then reshares the content, which then creates ‘downstream MSI’ through likes/reactions, comments, comment likes/reactions, and comment replies to and from the viewer's friends, who then continue to reshare the content and so on” (Haugen, 2021b, p. 2).

The optimization for MSI encourages the spread of misinformation, divisive political messages, and other problematic content. Because the algorithm only measures engagement and does not measure quality or attitude toward the content (e.g., via metrics like sentiment), optimizing for MSI pushes upsetting, hateful content that gets people to “engage” more: “Because MSI is designed to boost friend interactions, it doesn't value whether you’ll like a piece of content posted by the New York Times, Donald Trump, the Wall Street Journal, etc.” (Haugen, 2021b, p. 7). These revelations are consistent with a vast literature on how recommendation systems promote divisive content (e.g. Bakshy et al., 2015; González-Bailón et al., 2023; Huszár et al., 2022; Vosoughi et al., 2018). While Facebook depicts itself as a neutral platform immunized from liability for content posted by either “good” or “bad actors”, these revelations show that Facebook’s algorithms drive content. Facebook internal research shows “how outrage and misinformation are more likely to be viral” (Haugen, 2021b, p. 5), and provides “compelling evidence that our core product mechanics, such as virality, recommendations, and optimizing for engagement, are a significant part of why these types of speech flourish on the platform” (Haugen, 2021c, p. 7).

Misinformation, divisive political messages, and other problematic content keep users on the platform for longer, clicking on more pages and ads—in other words, content that increases MSI is good for Facebook’s business. The leaked documents show how “the more negative comments a piece of content instigates, the higher likelihood for the link to get more traffic … might reach the conclusion that darker, more divisive content is better for business” (Haugen, 2021b, p. 5). Even after employees made recommendations internally to address issues around content, Facebook executives rejected these recommendations due to a tradeoff with the company’s business: “Mark doesn't think we could go broad … We wouldn't launch if there was a material tradeoff with MSI impact” (Haugen, 2021b, p. 12).

Facebook’s front-stage data relation between a user and a so-called “bad actor” reduces a set of complex back-stage data relations structured by MSI. To illustrate this concretely, we can return to our example from above. A so-called “bad actor” such as the IRA, via a Facebook account posing as an American user, generates a Facebook post with a Pepe the Frog meme in a Facebook group of conservative American voters. Because that post will generate likes, clicks, and reshares, when that post is shared amongst many users within that group, and amongst each user’s network, and those users’ networks, and so on, it will be highly ranked in all those users’ feeds based on optimization for MSI. In the April 2018 testimony, Zuckerberg pointed out how “126 million people may have been served content from a Facebook Page associated with the IRA” (*S.Hrg. 115–683—Facebook, Social Media Privacy, and The Use and Abuse of Data*, 2018), emphasizing the IRA as the source of that content, but exactly how that content was able to reach 126 million people was precisely due to Facebook’s algorithmic optimization of MSI. Indeed, Facebook’s algorithms veer users *towards* groups or pages generated by “bad actors”. As the Facebook Papers illustrate, “Facebook has demonstrated via experiments using brand new test accounts how rapidly Facebook's algorithms can veer people interested in Conservative topics into radical or polarizing ideas and groups/pages, some demonstrating traits of Coordinated Inauthentic Behavior (CIB)” (Haugen, 2021a, p. 6). While Facebook focuses on targeting an easy “bad actor” scapegoat, the company fails to acknowledge that the platform's back-stage systems facilitate “abuse” in the first place.

Facebook’s front-stage reduction of these back-stage data relations is further demonstrated by the minimal effects that Facebook’s authenticity regulation efforts have had on these back-stage data relations. As the papers reveal, Facebook’s identity verification efforts fail to curb misinformation and hateful content: “Multiple offenders for Hate are frequently also multiple offenders for misinformation … We may be repeatedly applying authenticity verifications to some or many of these accounts to no effect … 99% of these user accounts remain active, and some of them have passed dozens of authenticity checks” (Haugen, 2021c, pp. 8–9).

Facebook purposefully hides these data relations from its project of regulating “bad actors”. Indeed, given these leaked internal documents reference Facebook’s *own research,* these documents illustrate how Facebook has self-consciously engaged in a strategic presentation of front-stage data relations while occluding back-stage data relations, in an effort to normalize a front-stage understanding of its platform. Since MSI is outside the user view of the platform, Facebook keeps MSI outside of the scope of action. As such, Facebook keeps data relations mediated by its recommendation systems—which drive the economic value of the platform—outside of the sphere of governance.

## Privacy Controls

### Governing Front-Stage “People-Centered Privacy”

This case details a governance project that Facebook enacted in response to the Cambridge Analytica controversy.^5^ In 2014, data of tens of millions of Facebook users were gathered through a personality quiz app created by Aleksandr Kogan; 270,000 users had installed the app, and by agreeing to the app’s terms of service, the app also accessed data of those users’ Facebook friends. Based on these data, Cambridge Analytica then trained predictive algorithms to micro-target political advertisements and messages to users—including tens of millions of Facebook users—in order to influence their voter behavior. When the scandal broke in March of 2018, there was widespread public outrage that Facebook users’ data were collected, shared, and used for psychographic targeting without users’ consent (Cadwalladr & Graham-Harrison, 2018; Gallagher, 2018; Hern, 2018; Rosenberg et al., 2018).

In response to the Cambridge Analytica scandal, Facebook made the following diagnosis: users didn’t have enough transparency and control over “bad actors” obtaining their information. At the April 2018 Senate Hearing, in response to questions raised about Facebook’s privacy policy, Zuckerberg said:

“... this gets into an—an issue that I—I think we and others in the tech industry have found challenging, which is that long privacy policies are very confusing. And if you make it long and spell out all the detail, then you're probably going to reduce the percent of people who read it and make it accessible to them. So, one of the things that—that we've struggled with over time is to make something that is as simple as possible so people can understand it, as well as giving them controls in line in the product in the context of when they're trying to actually use them, taking into account that we don't expect that most people will want to go through and read a full legal document.” (*S.Hrg. 115-683—Facebook, Social Media Privacy, and the Use and Abuse of Data*, 2018)

Here, Zuckerberg’s diagnosis reflects what Nissenbaum (2011) calls the “transparency paradox”: “If notice (in the form of a privacy policy) finely details every flow, condition, qualification, and exception, we know that it is unlikely to be understood, let alone read. […] An abbreviated, plain-language policy would be quick and easy to read, but it is the hidden details that carry the significance” (p. 36).

At the same time as pointing to inadequate transparency and controls, Zuckerberg painted Aleksandr Kogan and Cambridge Analytica as “bad actors” who improperly collected users’ data via Kogan’s app, which Zuckerberg referred to as an “abusive app” (Zuckerberg, 2018a). In recounting “exactly what happened with Cambridge Analytica,” Zuckerberg pointed to Kogan: “In 2013, a Cambridge University researcher named Aleksandr Kogan created a personality quiz app. It was installed by around 300,000 people who agreed to share some of their Facebook information as well as some information from their friends whose privacy settings allowed it.” Zuckerberg immediately followed up with how in 2014 Facebook had already tried “to prevent abusive apps […] like Kogan’s” (*S.Hrg. 115–683—Facebook, Social Media Privacy, and The Use and Abuse of Data*, 2018).

Framing the issue as users’ lack of control over “abusive apps” obtaining their information, Facebook proposed actions to increase transparency and control by the user. A few days after the Cambridge Analytica news stories broke in March 2018, Paul Grewal, VP & Deputy General Counsel highlighted that already in 2014 (“Suspending Cambridge Analytica and SCL Group From Facebook,” 2018), “we made an update to ensure that each person decides what information they want to share about themselves, including their friend list. […] Before you decide to use an app, you can review the permissions the developer is requesting and choose which information to share. You can manage or revoke those permissions at any time.” At the April 2018 Senate Hearing (*S.Hrg. 115–683—Facebook, Social Media Privacy, and The Use and Abuse of Data*, 2018), Zuckerberg proposed “building better controls” and making these controls more visible: “This week we started showing everyone a list of the apps you’ve used and an easy way to revoke their permissions to your data. You can already do this in your privacy settings, but we’re going to put it at the top of News Feed to make sure everyone sees it.” Here, Facebook takes actions clearly visible to the user: individual users can see and control the flow of personal information to apps they might use on the platform.

Facebook’s diagnoses and proposed solutions fall back on notice-and-consent (transparency-and-choice) practices within extant digital-privacy law. In response to the Cambridge Analytica controversy, a 2019 settlement with the U.S. Federal Trade Commission subjected Facebook to a $5 billion penalty and new privacy and data security obligations (U.S. Federal Trade Commission, 2019). The complaint alleged that Facebook violated a 2012 consent order with the FTC, which required “giving consumers clear and prominent notice and obtaining their express consent before sharing their information beyond their privacy settings” (U.S. Federal Trade Commission, 2012). After the settlement, Facebook doubled down on its diagnoses and proposed actions regarding user privacy controls.

In particular, Facebook expanded their efforts to increase transparency and control through “human-centered design.” In 2020 Facebook’s Chief Privacy Officer, Egan (2020) proposed a “solution to the transparency paradox”^6^ (p. 7): improving the user experience (UX) design of notices and controls. Egan (2020) explained, “in improving communication to people, more work is required to develop the right balance between making disclosures comprehensive and making them understandable” (p. 7). Transmuting the “transparency paradox” into a project of “human-centered design”, Egan aimed to create a user *experience* of control over their own information. Drawing upon extant practices in “design thinking”,^7^ Facebook developed an initiative of “people-centered privacy design”. Designating “privacy notifications as dynamic design challenges'', Egan (2020, p. 11) explained:

‘[h]uman-centric design’ or ‘people-centered design’ […] is an approach that focuses on the needs, concerns, and preferences of people at every step in the product design process […] If organizations consistently applied that same user-focused and iterative design process to designing privacy-related notices and controls, the results could very well be transformative.

Facebook deepened their work with TTC Labs—which stands for Trust, Transparency, and Control—working on “People-Centric Approaches to Notice, Consent, and Disclosure" (TTC Labs, 2019).

**Fig. 2:**
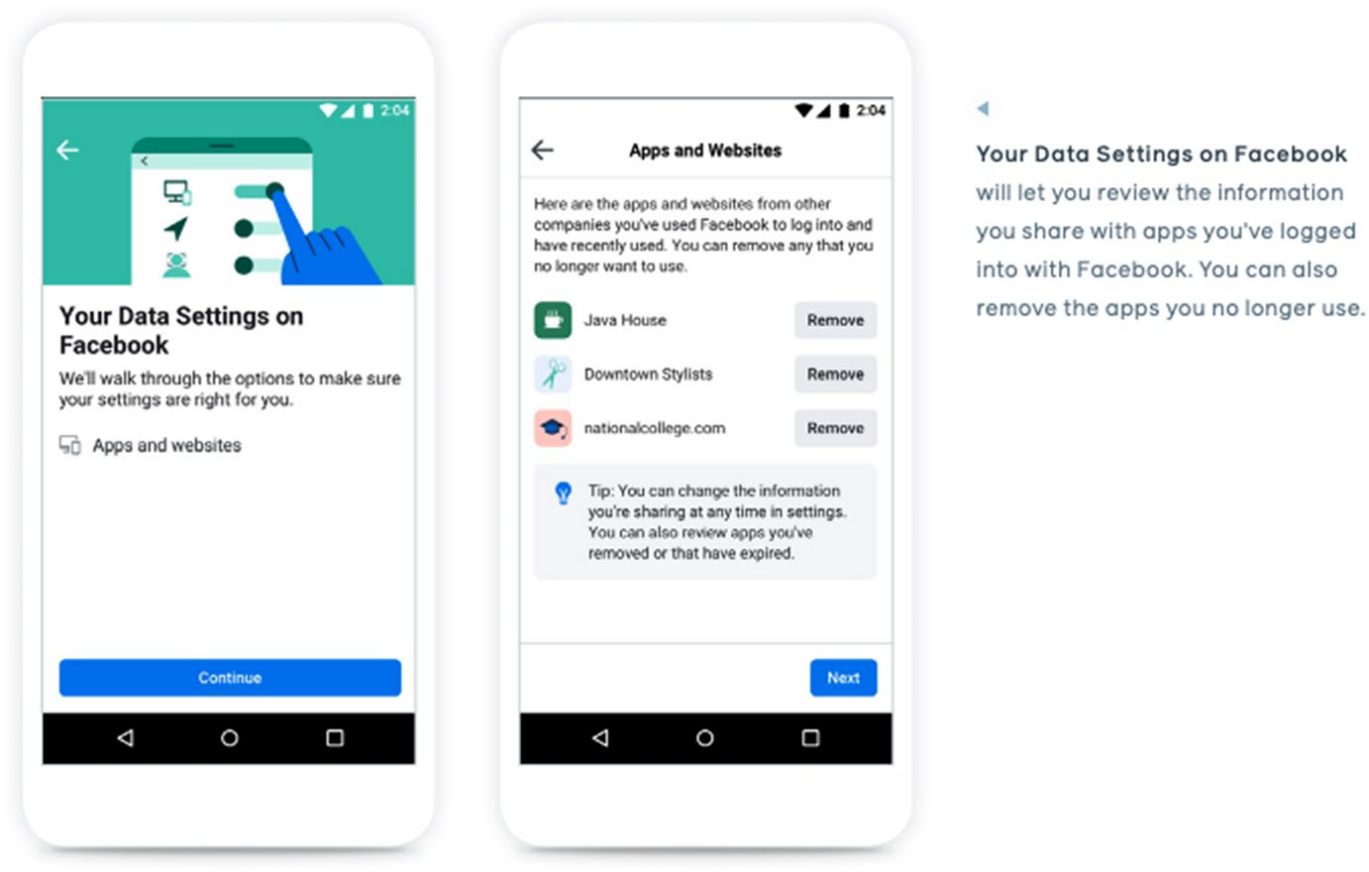
User Experience (UX) Design of “Your Data Settings on Facebook” (Egan, 2020, p. 24)

Facebook’s “people-centered privacy” project provides users with the capabilities to easily see and control the flow of personal information to third-party applications, advertisers, and other users. Egan presented an example of a user interface design that emerged from one of their “Design Jams” with TTC Labs (see Fig. 2, Egan, 2020, p. 24). On a user page called “Your Data Settings on Facebook”, the user is presented with a list of apps with icons and “Remove” buttons. The screen text says, “Here are the apps and websites from other companies you’ve used Facebook to log into and have recently used. You can remove any that you no longer want to use.” For example, the user can click to “Remove” an app called “Java House” from accessing the user’s Facebook information.

Facebook’s “people-centered privacy” project encompasses other user controls as well. Steve Satterfield—Privacy and Public Policy Director at Facebook—on a web page titled, “Personalized Advertising and Privacy Are Not at Odds” (Satterfield, 2020), explained, “For example, you can tap Why Am I Seeing This? on any ad in News Feed to get more information and control what you see going forward. […] From there, you also have easy access to controls, like Ad Preferences, which lets you manage the ads you see, learn more about how ads work and hide ads from specific advertisers or topics.” He further explained, “And our controls aren’t just for ads. We also offer tools like Manage Activity, Privacy Checkup and ways to view and download your information, so you can easily customize your overall experience on Facebook.” Indeed, on Facebook’s “Privacy Checkup” page, users can easily control “Who Can See What You Share”, toggling between “Only Me”, “Friends”, and “Public”.

This project aligned with Facebook’s broader efforts to “add more ways to interact privately with your friends, groups, and businesses” (Zuckerberg, 2021b): to impart a “sense of privacy and intimacy” in the service (Egan, 2020), or a “feeling of privacy” (Zuckerberg, 2021b). In a post titled, “A Privacy-Focused Vision for Social Networking”, Zuckerberg (2021b) spoke of a “future of the internet” shifting from “the digital equivalent of a town square” to “the digital equivalent of the living room”: “People should have simple, intimate places where they have clear control over who can communicate with them and confidence that no one else can access what they share.” As such, Facebook’s efforts to increase transparency and control over a user’s information cultivate a user *experience* of privacy. By making visible and persuasive modifications to the user interface, Facebook’s “people-centered privacy” project normalizes governance of the user experience of the platform.

### Occluding Back-Stage Data Relations

While Facebook’s privacy controls project constructs actionable front-stage data relations, this effort correspondingly relegates certain other data relations outside of the user experience to invisibility and inaction. The front-stage privacy controls obfuscate from users and regulators a highly complex network of data relations constituted by Facebook’s targeted advertising systems. Facebook’s targeted advertising systems have been described extensively (e.g., Vaidhyanathan, 2018); they are central to the mechanisms of surveillance capitalism (Zuboff, 2015) and platform capitalism (Srnicek, 2017). Facebook’s advertising auction, which sells space and time on Facebook pages to advertisers, serves as the company’s core business platform. Facebook developed a targeted advertising system to serve this ad auction.

Advertisements on Facebook can currently be purchased in three openly described ways (Meta for Business, n.d.)—through the ad auction and through two fixed price options—and all three of Facebook’s published advertising engines base part of their ad pricing models on Facebook’s predictions about users’ click-through rates. An ad that is more likely to be clicked can be sold for more, and Facebook also prefers to sell ads expected to be successful. As in traditional advertising (Brierley, 2005; Hodgson, 2023; Yankelovich, 1964), the most predictive features for targeting advertisements on Facebook are age, gender, and location (e.g., Karen et al., 2022). Advertisers can always select to target users based on these pieces of information (Meta Ads, n.d.).

Correspondingly, Facebook users are unable to opt out of these pieces of information being included in the Facebook ad engines. Facebook collects data on age, gender, and location from users outside of what Facebook designates as its privacy controls. Indeed, “[u]pon registration, most social media platforms require the disclosure of private information such as name, email address, gender and birth date” (DeNardis & Hackl, 2015, p. 763), and platforms also collect a wide range of metadata such as “IP address, unique hardware identifiers, software configurations, and a variety of locational indicators based on GPS, Wi-Fi, or cellular location” (DeNardis & Hackl, 2015, p. 764).

Facebook will not only collect information such as age and gender upon registration, but will also collect data on other features of the user based on population-level relevance. Further in the back stage are data relations assembling users with other users based on advertising-relevant features (cf., horizontal data relations, Viljoen, 2021), which allows Facebook to gather data on users similar to the user in question. For instance, even if you do not tell Facebook that you drink coffee, Facebook might infer that you are interested in coffee by a combination of what Facebook pages and posts you like, if your friends are interested in coffee, how old you are, and so forth. Facebook includes these inferred categories as further features that can be selected by advertisers for targeted advertising.

To illustrate concretely how Facebook’s front-stage data relations between users, apps, and advertisers reduce a set of complex back-stage data relations, we can return to our example from earlier. On the front stage, the user could click on a button to remove “Java House” from accessing the user’s Facebook information (see Fig. 2, Egan, 2020). This would mean that the company owning Java House can no longer view and download specific pieces of information about the user’s profile through the Java House app.

Nevertheless, any advertiser who wants to buy ads on Facebook can still indirectly access data on the user’s age, gender, and location. As such, the user in question can still be put into relation with “Java House” in the following manner: in the back stage, the data structuring the relation between the user and Java House are the user’s birthday, gender, location, and other features, as well as predicted click-through rate of any ads that the Java House company wants to buy. Additionally, the user is put into relation with other users of similar age, gender, and location—these users may have clicked on ads by Java House previously, or Java House may explicitly want to purchase ads targeted to this demographic.

These back-stage data relations allow Java House to target the user in question, as well as users similar in age, gender, and location, with advertisements. In other words, even if you have removed the Java House app, so that Java House cannot directly view your private data, and even if you have revoked permission from Facebook to target you ads based on the categories provided in the user controls, Java House can still target advertisements to your demographic data. As such, you may still receive advertisements from Java House because you fit the criteria for Java House’s targeted ad campaign, and because Facebook already retains data about you.

As of writing, currently Facebook’s front-stage privacy controls include opting out of categories such as “people with birthdays in February”, employer, job title, education, relationship status, and a variety of generic, inferred categories. However, these controls do not offer any option to opt out of targeted advertising completely or to opt out of being included in ads targeted based on age, gender, or location. Indeed, the front-stage Facebook privacy controls even include the following disclaimer in fine print:

“An advertiser can indicate that their ad should be shown to people who have certain information on their profile, such as a specific school or job title. You can choose whether this profile information can be used to show you ads in this way. This does not affect whether we can use this same profile information to add you to other categories or to otherwise help us personalize your ads. These settings don't change the information visible on your profile or who can use it.” (*Audience-Based Advertising*, n.d.)

Facebook purposefully hides these economically more relevant data relations from its project of platform governance. Since these data relations are outside the user view of the platform, Facebook keeps these data relations outside the scope of action.

Even though Facebook has since provided users with more controls on which information—such as their friends lists—to share with apps, Facebook nonetheless continues to collect data behind the scenes that could be harnessed by advertisers. Indeed, this functionality explains how data collected by Facebook—e.g., on age, gender, location, and other ad-relevant features—could then be used to train predictive algorithms, which could then be used to target other users deemed to share similar ad-relevant characteristics. Indeed, as Viljoen (2021, p. 605) explains, this “relational effect” is what allowed Cambridge Analytica to micro-target political advertisements to tens of millions of Facebook users, based on predictive algorithms trained off data collected on tens of millions of other Facebook users. As such, while Facebook has scapegoated “bad actors” such as Cambridge Analytica for “abusing” its platform, and has since foregrounded superficial options for users to avoid direct exploitation by known or suspected “bad actors”, Facebook continues to elide details on how its advertising engine works, as well as details on the data that drive the advertising—which continue to provide opportunities for “abuse” by “bad actors”. In so doing, the data relations mediated by Facebook’s targeted advertising system, which drive the economic value of the platform, are positioned outside the sphere of governance.

## Conclusions

How a platform is understood as a sociotechnical system matters for how a platform is governed (Ananny, 2016; Ananny & Crawford, 2018; Gillespie, 2010, 2023; Gorwa, 2019; Napoli & Caplan, 2017). As Gilespie (2010) astutely described:

As society looks to regulate an emerging form of information distribution, be it the telegraph or radio or the internet, it is in many ways making decisions about what that technology is [...] This is a semantic debate as much as anything else: what we call such things, what precedents we see as most analogous and how we characterize its technical workings drive how we set conditions for it. (p. 355-356)

Controversies such as Cambridge Analytica and Russian Interference in the 2016 U.S. Presidential Election have provided opportunities for reflexivity amongst users, regulators, and publics on the terms by which to understand platforms and how to regulate them. In response to these controversies, platform companies such as Facebook have doubled down on projects of privatized governance pertaining to content moderation and transparency-and-control. We have shown how Facebook has attempted to achieve closure, through techniques of stage management, on how platforms are understood and governed.

Revelations about the back stage of Facebook’s platform—via whistleblower leaks such as the Facebook Papers, investigative journalism, and critical scholarly research—show how Facebook *self-consciously* manages what audiences of users and regulators are capable of perceiving. Taking advantage of the front-stage, back-stage division of its platform, Facebook strategically constructs front-stage data relations for governance. These platform governance projects propose to act upon information that users can see from other users—whether that is content that users can see from “bad actors”, or information that others can see about oneself. Facebook renders the user experience actionable for governance, while relegating back-stage data relations—central to the platform’s economic value—to invisibility and inaction. As such, Facebook’s efforts foreclose governance of the political economy of platforms.

Facebook’s two projects of authenticity regulation and privacy controls still persist today. In order to address polarization and misinformation on its platform, Facebook continues to rely on taking down accounts of “bad actors”. In August 2023, Meta stated it had identified and taken down large-scale operations of inauthentic accounts connected to a Russian influence operation (impersonating European news outlets) aiming to erode support for Ukraine (Bond, 2022), as well as inauthentic accounts spreading pro-China messages and attacking U.S. and European foreign policy (Bond, 2022).

Facebook’s current project of building the “Metaverse”—and rebranding as Meta—repackages their “people-centered privacy” project. Highlighting on its page, “Responsible innovation starts with privacy” (Metaverse, n.d.), Meta emphasizes, “It’s our responsibility to create default privacy settings that put people first, but also to give people tools to manage their privacy, their way. That means designing controls for transparency and ease of use.” Narrowing in once again on the user experience of the platform, Meta re-produces a front-stage data relation between the user and other users. In an introductory video to the Metaverse, Zuckerberg highlights the user experience of privacy, echoing his prior framing of Facebook as a private living room: “You’ll get to decide when you want to be with other people, when you want to block someone from appearing in your space, or when you want to take a break and teleport into a private bubble to be alone” (Meta, 2021).

In the midst of ongoing contestation over how to understand and govern platforms, Facebook’s governance projects still attempt to reify a front-stage understanding, reinforcing information asymmetry. As social media companies continue to perform platform governance projects following controversy, our paper invites reflection on the politics of these projects. Despite what platform companies may want audiences to believe, as Gillespie (2023) aptly reminds us, “What social media is remains unsettled.” We aim to unsettle any stabilizations in front-stage understandings that platform companies might have achieved. We have shown how standpoint—in this context, within the user view or outside the user view of the platform—configures understanding (Jasanoff, 2017). By showing how a platform governance project can construct a more limited view, and by interrogating the division between the front stage and back stage, we aim to expand the boundaries of platform governance. Ananny and Crawford (2018) argue for understanding an algorithmic system as a distributed, relational system of human and non-human actors, which would require governance that sees *across* the system. As such, we open space for seeing *across* a platform—that is, across both the front-stage and back-stage data relations—in the pursuit of governing platforms as sociotechnical systems.

We contribute to collective destabilization of Facebook’s front-stage performances that attempt to naturalize their back-stage operations. In response to continuous controversies, users and regulators have sparked contestation and debate regarding how to govern platforms, moving towards frameworks that press for regulating platforms as commercial entities—that is, for taking action on the back-stage data relations. In his dissenting statement to the 2019 FTC Order, Commissioner Rohit Chopra identified Facebook’s behavioral surveillance as the “root cause” of the Cambridge Analytica controversy. He urged:

It is now more important than ever for global regulators and policymakers to address the threats posed by behavioral advertising. Absent an effective framework, we need to ask whether we need a moratorium on behavioral advertising by dominant platforms. We should question whether the traditional approach to privacy protection could ever fix these flaws. (Chopra, 2019)

Chopra, now director of the Consumer Financial Protection Bureau, continues to scrutinize the inadequacy of the 2019 FTC settlement, as the settlement did not materially address Facebook’s business practices based on surveillance and targeted advertising (Chopra, 2022). As of writing, the current Banning Surveillance Advertising Act, introduced by Congresswoman Anna G. Eshoo, Congresswoman Jan Schakowsky, and Senator Cory Booker, proposes to ban targeted ads based on personal data on protected characteristics (e.g., race, gender, religion), and personal data purchased from third parties (*Eshoo, Schakowsky, Booker Introduce Bill to Ban Surveillance Advertising*, 2022). Along similar lines, legal scholar Julie Cohen has argued for framing platforms as companies rather than as neutral information intermediaries. This means understanding “what platforms are not: they are not publishers, nor are they public fora […] Platforms are private, for-profit entities that operate as central nodes in the contemporary personal data economy” (Cohen, 2020, p. 656). In the summer of 2020, civil rights organizations initiated the #StopHateForProfit campaign, urging companies to stop their advertising on Facebook in order to protest Facebook’s handling and proliferation of hateful content (Heilweil, 2020). In response to Frances Haugen’s testimony, nearly fifty civil society organizations signed a petition, “Here’s How We Stop Facebook” in an effort to “Tell lawmakers to investigate Facebook and pass a real data privacy law that ends their harmful business model forever” (*Here’s How We Stop Facebook*, n.d.). As users, regulators, and broader publics engage in these reflexive efforts, our analysis contributes to this collective re-claiming on how platforms—and their co-constitutive data relations—should be understood and governed.

## Footnotes

1 In October 2021, weeks after the Facebook Papers leak, Facebook Inc. announced its name change to Meta Platforms (“The Facebook Company Is Now Meta,” 2021). The company’s name did not officially change until June 9, 2022 (Curry & Powell, 2022). In this paper, we refer to the company and platform in question as Facebook. This is for two reasons. First, our principal case studies pertain to the Russian Interference and Cambridge Analytica controversies surrounding the 2016 U.S. Presidential Election, and platform governance projects enacted by the company in response to these controversies, which occurred before the company’s name change to Meta. Much of the evidence for these two cases, such as from the Facebook Papers leaked by Frances Haugen, refer to the company and platform as Facebook. Second, the platform governance projects that we investigate were, and continue to be, implemented in the Facebook social media platform, which has retained the name Facebook under the umbrella company of Meta Platforms.

2 Despite much criticism regarding the notice-and-consent privacy regime (see Viljoen, 2021 for review), this remains the status quo mode of data governance in the United States.

3 We adopt the term “authenticity regulation” from Haan (2020).

4 Within the context of our analysis, social media platforms, we define data as digital information. We define a data relation as a social, economic, or political relation between entities mediated by a digital environment (cf., Viljoen, 2021; Couldry & Mejias, 2019). We do not take a data relation to be singular, static, or universal. Our conception of data relations embraces a multiplicity of relations mediated or unmediated by data (cf., Marres, 2017).

5 This controversy has been described extensively elsewhere (e.g. Cadwalladr & Graham-Harrison, 2018; Confessore, 2018; Krogerus, 2017; Meredith, 2018; Rosenberg et al., 2018); we briefly summarize relevant details here.

6 Egan explicitly cites Nisssenbaum (2011) when referring to the “transparency paradox.” This is quite an incredulous instance of capture, as the Nissenbaum (2011) article appealed to by Egan clearly argues against proposals that attempt to improve notice-and-consent, and argues in favor of alternative approaches rooted in “contextual integrity” (see Nissenbaum, 2011 for details).

7 Design thinking “is a term popularized in Palo Alto [Silicon Valley] by the design firm IDEO and Stanford University through forums like TED, BusinessWeek, and Harvard Business Review” (Irani, 2018, p. 3). Design thinking proposed starting with understanding from the user’s needs, preferences, and perspectives, and incorporating the user in subsequent steps in designing the product or service (McGrath, 2020; IDEO, n.d.).
